# Effects of bottom trawling on fish foraging and feeding

**DOI:** 10.1098/rspb.2014.2336

**Published:** 2015-01-22

**Authors:** Andrew Frederick Johnson, Giulia Gorelli, Stuart Rees Jenkins, Jan Geert Hiddink, Hilmar Hinz

**Affiliations:** School of Ocean Sciences, Bangor University, Menai Bridge, Anglesey LL59 5AB, UK

**Keywords:** fishing impacts, bottom trawl fishery, diet analysis, foraging behaviour, predator–prey interaction, ecosystem-based fisheries management

## Abstract

The effects of bottom trawling on benthic invertebrates include reductions of biomass, diversity and body size. These changes may negatively affect prey availability for demersal fishes, potentially leading to reduced food intake, body condition and yield of fishes in chronically trawled areas. Here, the effect of trawling on the prey availability and diet of two commercially important flatfish species, plaice (*Pleuronectes platessa*) and dab (*Limanda limanda*), was investigated over a trawling intensity gradient in the Irish Sea. Previous work in this area has shown that trawling negatively affects the condition of plaice but not of dab. This study showed that reductions in local prey availability did not result in reduced feeding of fish. As trawling frequency increased, both fish and prey biomass declined, such that the ratio of fish to prey remained unchanged. Consequently, even at frequently trawled sites with low prey biomass, both plaice and dab maintained constant levels of stomach fullness and gut energy contents. However, dietary shifts in plaice towards energy-poor prey items were evident when prey species were analysed individually. This, together with a potential decrease in foraging efficiency due to low prey densities, was seen as the most plausible cause for the reduced body condition observed. Understanding the relationship between trawling, benthic impacts, fish foraging and resultant body condition is an important step in designing successful mitigation measures for future management strategies in bottom trawl fisheries.

## 1. Introduction

Demersal fisheries using otter and beam trawls are widespread over shelf seas, and typically use heavy ground ropes and chains to drive fish and crustaceans from the seabed into nets. Physical disturbance from such fisheries can cause significant changes in benthic invertebrate abundance, biomass, production and species richness [1–4]. While many invertebrate species are negatively affected by demersal trawling, other more resilient species may show little response [5], resulting in anthropogenically modified benthic species assemblages. Changes in benthic composition may subsequently affect the quality and quantity of prey for demersal, benthivorous fish species [6–9]. The general response to a reduction in benthic biomass as a consequence of demersal trawling is thought to decrease the overall carrying capacity for demersal fishes through reduced prey availability [10]. The response of individual fish species will, however, depend on the susceptibility of its prey to fishing disturbance [8]. Negative effects can be expected if fishing leads to a reduction in the biomass of preferred prey [1,7,11], whereas no effect or a positive effect may be expected if the prey is not influenced or benefits from the fishing activity, e.g. (if its prey profits from scavenging on organisms that are damaged by the trawl or competitive release from trawl-sensitive competitors) [12–14].

The response of individual fish species could also be shaped by their feeding strategy and the prey species they preferentially feed on. For example, fish species that target a wide prey spectrum may be less affected by an overall reduction in the abundance of vulnerable benthic invertebrates as they are likely to be able to supplement the loss of vulnerable prey types by switching to those less vulnerable or those prey items whose availability increases following a trawl pass [10,12]. Trawling may, however, have a strong negative effect on the foraging success of specialized feeders, which have been shown to be particularly vulnerable to changes in prey availability [15]. It is clear from a number of empirical and modelling studies that fishing-induced changes in benthos and consequent changes in fish food availability may have important impacts on fish body condition [1,7,11] and possibly population levels [11,16]. Off the eastern Scotian Shelf, Choi *et al.* [7] linked significant declines in the condition of ground fishes to the reduction in benthic food resources on heavily trawled fishing grounds. In the Celtic Sea, significant declines in length-at-age of plaice (*Pleuronectes platessa*) were found with increasing trawl frequencies over gravel habitats dominated by fragile benthic organisms, while an increase in length-at-age of plaice was detected over sandy habitats that tend to be dominated by less vulnerable species [11]. Van Denderen *et al.* [8] concluded in a modelling study that the effect of trawling on fish populations was highly dependent on the vulnerability of prey to trawling, the strength of competition between prey and non-prey organisms, and the extent to which the system was characterized by bottom-up or top-down control. Fishing resulted in higher fish yields and increased persistence when the benthos representing best-quality fish food was also much more resistant to trawling than non-preferred prey. These positive effects occurred in bottom-up controlled scenarios where fish feeding had only limited impact on benthic biomass. By contrast, fishing led to lower yields and fish persistence in all scenarios (top-down and bottom-up controlled systems) when high-quality preys were negatively affected by trawling.

Despite the advances made in empirical and modelling studies, there is still a lack of mechanistic understanding of how trawling-induced changes in benthic invertebrates determine fish condition, and ultimately population parameters through fish diet. To date, few studies have analysed the relationships between prey resources and fish populations at the fishery spatial scale [17], and there have been no simultaneous examinations of the effects of bottom trawling on prey availability, fish stomach contents and fish condition. Here, we examine how the feeding of two commercially important flatfishes, plaice (*P. platessa*) and dab (*Limanda limanda*), was affected by chronic trawling on a *Nephrops norvegicus* fishing ground in the Irish Sea, UK. Hiddink *et al.* [1] found that the condition of plaice on this fishing ground was negatively related to trawling intensity, while the condition of dab showed no such relationship. Using detailed stomach content analysis of those fish sampled by Hiddink *et al.* [1], we examine how differences in the feeding ecology of these species causes different dietary responses to trawling and how these may be linked to overall differences in body condition.

Plaice predominantly target infaunal prey of limited mobility (such as polychaetes and bivalves) [16,18] through a well-developed suction capability, horizontal mouth/head down foraging position and large olfactory bulb [19,20]. By contrast, dab are large-eyed visual, opportunistic predators, suited to feeding on prey items found on the surface of the sea bed [20–22], targeting primarily mobile prey such as crustaceans, while being comparatively ineffective infaunal feeders [16,23]. Given the difference in feeding ecology of the two flatfish species studied, it was hypothesized that the reduced condition of plaice in response to trawling was related to decreased abundance/availability of its narrow prey spectrum at frequently trawled sites. Hence, we expected that plaice stomach contents would show a reduction in biomass and energy content with increasing trawling frequency. By contrast, it was predicted that the more opportunistic feeding strategy of dab would be more readily adapted to perturbations in the availability of its prey, and consequently stomach content biomass and energy content would be unaffected by trawling. By examining the feeding of demersal fish species across a chronic trawling intensity gradient, this study provides an important step towards a mechanistic understanding of how such fishing activity can indirectly affect fish populations mediated through diet.

## 2. Material and methods

### (a) Study area

**Figure 1.**
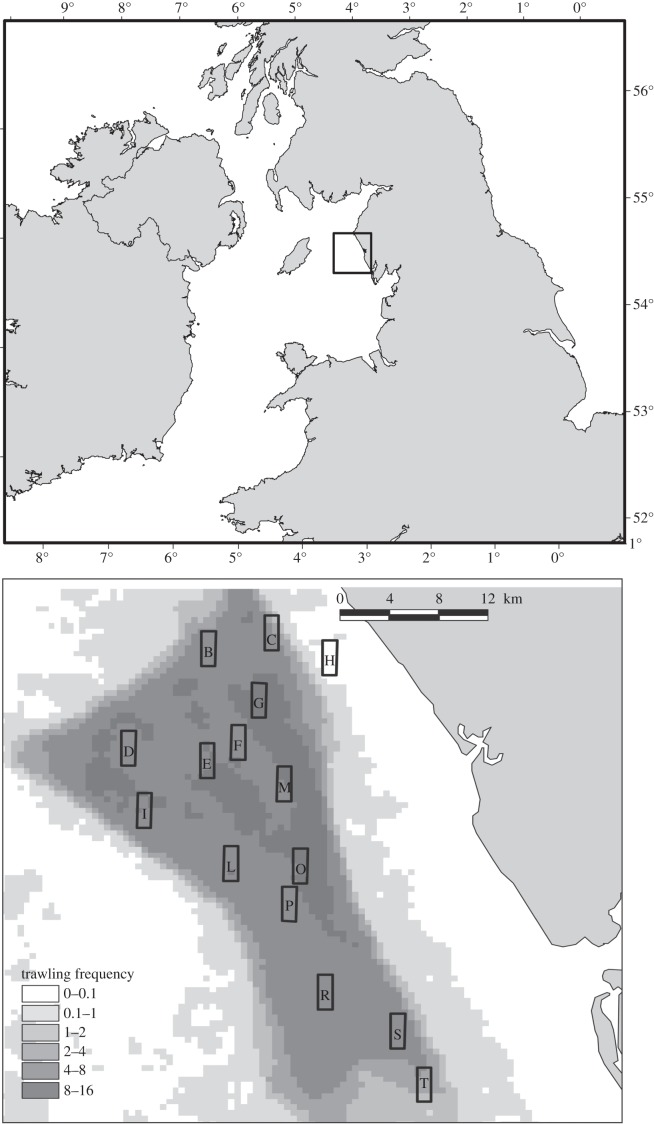
Sampling stations and the distribution of bottom trawl frequency (year^−1^) from 2004 to 2008 in the study area (as in [1]).

The effect of trawling on fish diets was investigated over a gradient of commercial bottom trawling effort in an area of otherwise homogeneous environmental conditions, off the Cumbrian coast (UK), in the northeastern Irish Sea (figure 1). This area is subjected to a wide range of trawling frequencies (between 0.5 and 11.9 trawl passes per year; see the electronic supplementary material, table A1), with a peak in activity from spring to early summer [24]. The fishery targets *N. norvegicus*, and commercial trawl frequencies were calculated using fishery protection over-flight observations and Vessel Monitoring System data (figure 1). The area is characterized by low-energy hydrodynamic conditions, and consequently the substratum comprises mostly fine sand and muddy sediments [24].

### (b) Sampling fish and invertebrate populations

Fifteen stations were selected for sampling within the study area, each comprising a 1 × 2 km box. Locations of sampling sites were chosen to cover the widest range of trawl frequencies while keeping other environmental conditions as constant as possible [24]. Fish and benthic infauna were sampled at each station in June 2009.

The demersal fish community at each station was sampled by conducting two 30 min tows at three knots using a rock-hopper otter trawl (distance across mouth of the net 16 m, head line height 3 m, 82 mm diamond mesh cod-end). Plaice and dab of total body length (TBL) 182–299 mm and 168–274 mm, respectively, were selected for stomach content analysis. These sizes were selected to ensure that the mean mouth gapes (calculated as the perimeter of an ellipse [25]) of the plaice and dab groups overlapped, meaning differences in prey sizes consumed between the two species were due to selectivity and not mouth gape constraints (electronic supplementary material, figure A1). These size ranges also minimized the likelihood of incorporating ontogenetic changes in diet [26]. Within these size ranges, stomachs of two individuals from each 10 mm size class of each species were extracted and stored in 8% buffered formalin for processing.

The mass of the entire stomach (whether full or empty) and total mass of prey contents were recorded after blotting. Prey items were then separated, identified to the highest taxonomic resolution possible (at least genus), counted, rated according to digestive stage (1 = fresh, 2 = partial, 3 = well digested), weighed and measured (as described by Johnson *et al.* [25]). Only prey items of digestive stages 1 and 2 were included in analyses of prey species biomass and prey size, because individuals at digestive stage 3 provided inaccurate estimates of biomass and size due to increased liquid retention associated with more advanced digestive states. In total, 414 plaice and 575 dab stomachs were analysed. Plaice from station I were not considered in the analysis as only one individual in the given TBL range was caught. All other stations included at least 11 individuals of plaice and dab (see the electronic supplementary material, table A1).

Benthic infauna were sampled by taking five 0.1 m^2^ Day grabs at haphazard locations in each station box. Samples were sorted over a 1 mm sieve and preserved in 4% formalin, and later identified to the highest taxonomic resolution possible. The wet biomass of each individual organism was measured after blotting. Results from the five individual grabs were pooled before statistical analyses to provide an estimate of faunal abundance and biomass for each station.

### (c) Data analysis: dietary descriptors of plaice and dab

Several dietary descriptors were used to quantify differences in feeding strategies between plaice and dab over the trawling intensity gradient. Differences in prey preferences were investigated using Chesson's index (standardized forage ratio) [27,28]. Chesson's index shows preferred prey types by comparing the availability of a prey item in the environment with the presence of the prey in stomach contents. Stomach contents from all stations were combined, and only those prey occurring more than 10 times in the diet of plaice and dab across all sites (herein referred to as common prey species) were included in the calculation of this index; these species accounted for 91% and 89% of the diets by weight of plaice and dab, respectively. The index (*α*_a_) ranges between 0 (complete avoidance) and 1 (exclusive feeding), and was calculated for each of the common prey species analysed [29,30] as
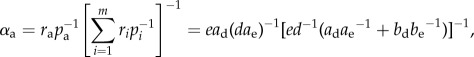
where *a*_d_ is the number of prey animals of species *a* in the predator's diet, *b*_d_ is the number of all other prey animals in the diet, *a*_e_ is the number of prey animals of species *a* in the environment, *b*_e_ is the number of all other prey animals in the environment, *d* is the total number of all animals in the diet, *e* is the total number of all animals in the environment, *r*_*a*_ is the proportion of prey species *a* in the diet and *p_*a*_* is the proportion in the environment*.* Preferential prey selection (when a prey is taken by the predator in higher proportions than it exists in the environment) occurs when *α*_*a*_ > 1/*m*, where *m* is the total number of different prey species in the stomachs of the predator.

Levins's niche breadth [31] was calculated to determine the range of prey (species and sizes) targeted by plaice and dab using the formula
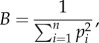
where *p_i_* is the relative occurrence of prey taxon *i* in a given species's diet. The index describes the amount of potential prey resources available to a predator in an environment with a known prey community [32]. Increasing values of *B* indicate more prey options available to the predator.

In order to determine whether the size of prey relative to the mouth size was significantly different between plaice and dab, the ratio between prey width and mouth width (PW : MW) was compared using an independent-samples *t*-test, using the mean values from each site as replicates. To investigate differences in energy content of the prey species consumed, the mean energy content per stomach was calculated using biomass conversion factors [33] (electronic supplementary material, figure A2). The level of stomach fullness to which plaice and dab fed at each site was calculated as the mean stomach fullness as a percentage of body biomass (Hyslop's index) [34]. The overall stomach energy contents and stomach fullness of plaice and dab were also compared using independent sample *t*-tests, using the mean values from each site as replicates.

### (d) Data analysis: effect of trawling on prey and diet compositions of plaice and dab

Differences in prey species composition between the diets of plaice and dab over the trawling gradient were explored using multivariate statistics in the PRIMER (v. 6) software package with PERMANOVA extension [35]. An ANOSIM test was undertaken to see if there was a significant difference in the diet composition (prey abundances in stomachs) of plaice and dab and a distance-based linear model (DISTLM) was used to determine whether diet was related to trawl frequency. All multivariate data were square-root-transformed to down-weigh the contribution of quantitatively dominant species.

Ordinary least squares (OLS) regressions were used to determine if trawling frequency reduced the total prey abundance, total biomass and individual total biomasses of the top 90% (by abundance) of prey species in the environment. The response of the total abundance and total biomass of prey species in the stomachs as well as the response of each of the univariate dietary descriptors (Hyslop's fullness, Levins's niche breadth, stomach energy content and PW : MW) was also analysed using OLS regressions. Finally, a per species analysis used OLS regressions to analyse the effect of trawl frequency on the mean body size, total biomass and abundance of each of the common prey species in the stomachs of plaice and dab. If trawling reduces the abundance of fish at the same rate as the reduction in the availability of their food sources, the amount of food that is available to each fish may not change even where the total prey biomass is strongly reduced. We therefore also examined how trawling affected the ratio of the biomass of prey in the environment to the biomass of fish in the environment.

All univariate response variables were log_10_-transformed (except indices, e.g. Levins's, Hyslop's and Chesson's) before statistical analysis to approximate normality and homogenize variances. When the response to trawling of multiple species from the same samples is tested, the chance of Type I errors increases. To control the false discovery rate (FDR) associated with multiple hypothesis testing, a post hoc threshold *α*-value was calculated as described by Benjamini & Hochberg [36]. This gave conservative *α*-values for each set of multiple tests performed. It should be noted that although FDR corrections reduce the chance of Type I errors during multiple testing, they increase the possibility of generating false negatives (Type II errors). Original *p-*values are reported as well as significance after FDR correction. Only those regressions that were significant after FDR correction are plotted in figures.

## 3. Results

### (a) Comparison of dab and plaice diet

**Figure 2.**
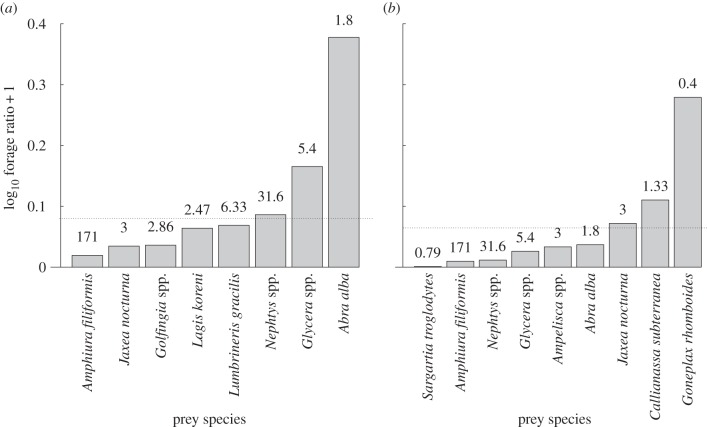
Chesson's index (prey preference) for the most common prey species (those occurring more than 10 times in the stomachs of dab and plaice, respectively) combining data from all stomachs analysed from all sites. Broken lines mark the threshold of significant preferential selection *m* (plaice *m* ≥ 0.083, dab *m* ≥ 0.067). Above the broken line denotes active selection and below it denotes no preferential selection. Numbers above each bar denote the mean density of each prey in the environment per m^2^.

The ANOSIM routine identified that the diet composition of the two flatfish species were significantly different (*p* = 0.001, global *R* = 0.731). The Chesson's index (*α*_*a*_) indicated that plaice had a preference for bivalves (*Abra alba*) and polychaetes (*Glycera* spp. and *Nephtys* spp.), while dab primarily favoured crustacean species (*Goneplax rhomboides*, *Calianassa subterranea* and *Jaxea nocturna*; figure 2). Presence of these species in the stomachs of dab was generally as whole chelae only. These were totally absent from the stomachs of plaice. Dab had significantly higher stomach fullness than plaice (*t* = 3.512, d.f. = 27, *p* < 0.001), with the stomach contents of dab containing significantly more energy per gram of fish (on average six times higher) than plaice (*t* = −11.089, d.f. = 401, *p* < 0.001; dab: 19.18 ± 2.57 versus plaice: 3.22 ± 0.54 J g^−1^ of fish, mean ±s.e.). Dab also had a significantly higher PW : MW ratio (*t* = −5.821, d.f. = 18.08, *p* < 0.001) than plaice (0.519 ± 0.033 versus 0.319 ± 0.024) and a significantly greater Levins's niche breadth (*t* = −2.069, d.f. = 27, *p* = 0.05).

### (b) Effect of trawling on prey and diet compositions of plaice and dab

**Figure 3.**
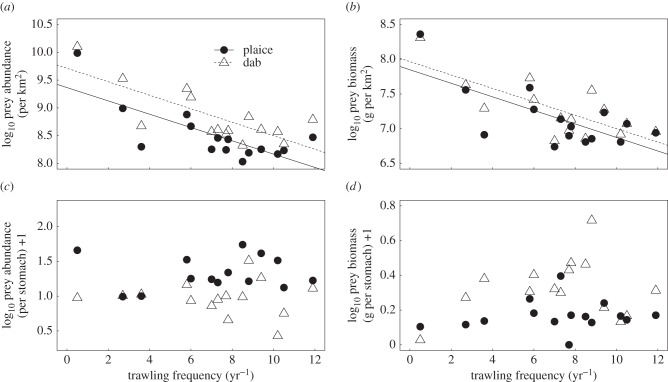
The relationship between bottom trawling frequency and (*a*) abundance of prey species, (*b*) biomass of the prey species in the environment, (*c*) mean prey abundance and (*d*) mean prey biomass in the stomachs of plaice and dab. Fitted lines represent OLS regressions. Each point represents the mean value per site.

The response of the biomass of all individual prey species in the environment to trawling is given in the electronic supplementary material, table A2. Of the 24 infaunal species in the environment, six showed significant negative relationships with increasing trawl frequency. Only one species, the bivalve *Corbula gibba*, showed a significant positive relationship with trawling, but it was not an important species in the diets of plaice or dab. The abundance and biomass of the common prey species of plaice (abundance: *R*^2^ = 0.57, *F*_1,13_ = 17.25, *p* < 0.001, biomass: *R*^2^ = 0.51, *F*_1,13_ = 13.09, *p* < 0.001) and dab (abundance: *R*^2^ = 0.58, *F*_1,14_ = 17.98, *p* < 0.001, biomass: *R*^2^ = 0.55, *F*_1,14_ = 15.77, *p* < 0.001) in the environment showed significant negative relationships with increasing trawl frequency (figure 3*a*,*b*).

**Figure 4.**
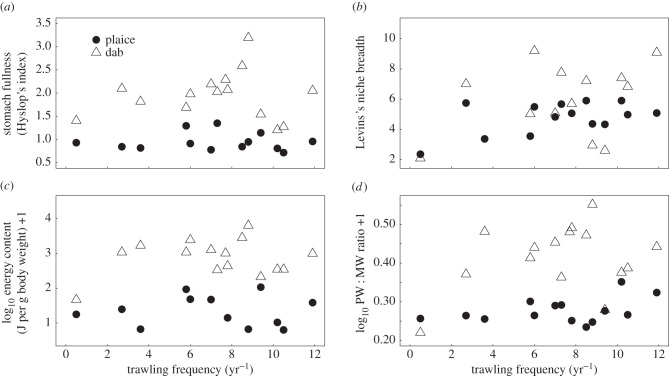
The relationship between bottom trawling frequency and (*a*) stomach fullness, (*b*) Levins's niche breadth of the prey species in the stomachs, (*c*) energy content of stomach contents and (*d*) the mean ratio of prey width to mouth width of plaice and dab. Fitted lines represent OLS regressions. Each point represents the mean value per site.

**Figure 5.**
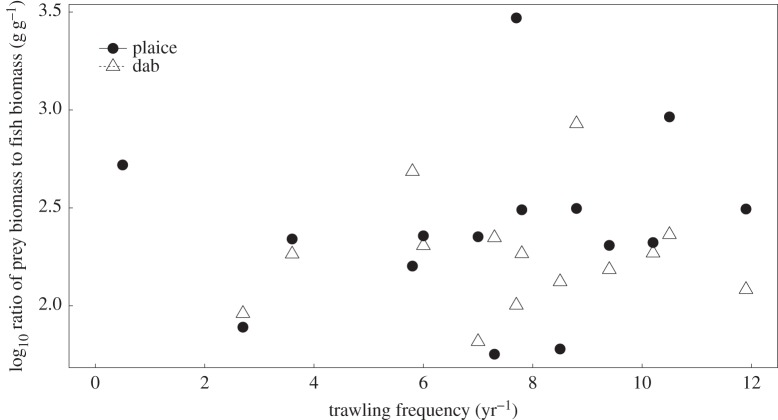
The relationship between bottom trawling frequency and the ratio of prey biomass to fish biomass (g g^−1^). Each point represents the mean value per site.

The DISTLM analysis indicated that trawl frequency had a significant effect on the diet composition of plaice (*F*_1,13_ = 3.71, *p* = 0.007) and dab (*F*_1,14_ = 2.71, *p* = 0.026). There was no significant relationship between trawling frequency and the abundance of prey items in the stomachs of plaice (*F*_1,13_ = 0.056, *p* = 0.817) or dab (*F*_1,14_ = 0.091, *p* = 0.769), nor the total biomass of prey items in the stomachs of plaice (*F*_1,13_ = 0.561, *p* = 0.468) or dab (*F*_1,14_ = 0.611, *p* = 0.448; figure 3*c*,*d*). Trawling also had no significant effect on the stomach fullness of plaice (*F*_1,13_ = 0.004, *p* = 0.949) or dab (*F*_1,14_ = 0.098, *p* = 0.759; figure 4*a*). The Levins's niche breadth in the diets of dab showed no relationship with trawling (*F*_1,14_ = 0.009, *p* = 0.926), while for plaice there was a marginally non-significant positive relationship (*R*^2^ = 0.278, *F*_1,13_ = 4.616, *p* = 0.06; figure 4*b*). Neither the stomach energy contents per gram of body weight of individual fish (plaice: *F*_1,13_ = <0.001, *p* = 0.981, dab: *F*_1,14_ = 0.553, *p* = 0.47; figure 4*c*) nor the mean prey width to mouth ratio of the plaice or dab showed significant relationships with increasing trawl frequency (plaice: *F*_1,13_ = 0.184, *p* = 0.675, dab: *F*_1,14_ = 1.563, *p* = 0.233; figure 4*d*). The ratio of prey biomass to fish biomass in the environment did not change significantly with increasing trawl frequency for either plaice (*F*_1,13_ = 0.219, *p* = 0.648) or dab (*F*_1,14_ = 3.062, *p* = 0.104; figure 5).

**Figure 6.**
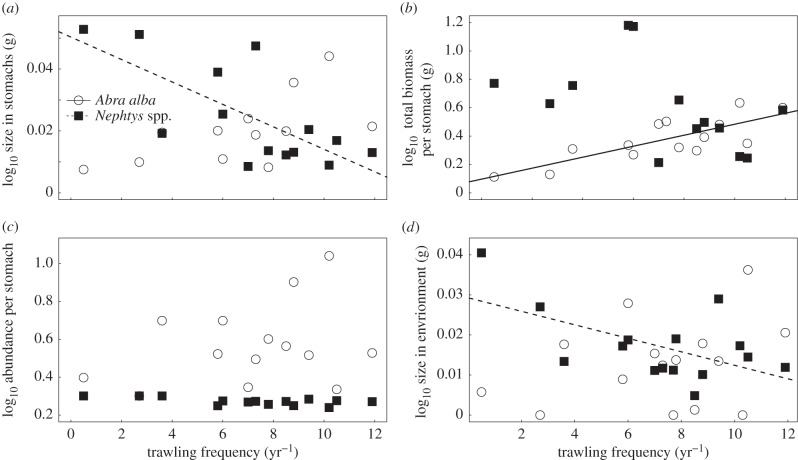
The relationship between trawl frequency and (*a*) prey size in the stomachs, (*b*) the total prey biomass in the stomach contents, (*c*) the number of prey per stomach and (*d*) the body size in the environment for *A. alba* and *Nephtys* spp*.* Lines represent OLS regressions after FDR correction. Each point represents the mean value per site.

Both *A. alba* and *Nephtys* spp. were preferentially selected by plaice in the study area, and these were the only prey species that showed significant responses to trawling in the stomachs. Figure 6 therefore only displays the responses of these two species. As trawling frequency increased, the body size of *Nephtys* spp. in the stomach contents of plaice decreased significantly (*R*^2^ = 0.477, *F*_1,13_ = 12.83, *p* = 0.004; figure 6*a*; electronic supplementary material, table A3). The total biomass of *Nephtys* spp., however, did not decrease with trawl frequency (*F*_1,13_ = 1.343, *p* = 0.269; figure 6*b*; electronic supplementary material, table A3). There was no significant decline in the number of *Nephtys* spp. in the stomachs of plaice after FDR correction (abundance: *R*^2^ = 0.384, *F*_1,13_ = 7.477, *p* = 0.018; figure 6*c*; electronic supplementary material, table A3).

The body size (*R*^2^ = 0.376, *F*_1,13_ = 5.99, *p* = 0.034) of *A. alba* in the stomachs of plaice did show an increasing trend with trawling intensity, but this was not significant after FDR correction (figure 6*a*; electronic supplementary material, table A3). The total biomass, however, increased significantly (*R*^2^ = 0.63, *F*_1,13_ = 20.83, *p* < 0.001) with increased trawling (figure 6*b*; electronic supplementary material, table A3), while at the same time the number of *A. alba* in the stomach contents showed no significant change (*F*_1,13_ = 1.256, *p* = 0.284; figure 6*c*; electronic supplementary material, table A3), reiterating the initially apparent increase in body size with trawl frequency.

In the environment, the body size of *Nephtys* spp. (*R*^2^ = 0.33, *F*_1,13_ = 6.46, *p* = 0.024) decreased significantly with increasing trawling, whereas that of *A. alba* (*F*_1,13_ = 1.264, *p* = 0.281) showed no significant relationship with trawling (figure 6*d*). Neither the abundance of *Nephtys* spp. (*F*_1,13_ = 1.367, *p* = 0.263) nor that of *A. alba* (*F*_1,13_ = 0.332, *p* = 0.574) in the environment showed a significant relationship with trawling. Trawling had no significant effect after FDR correction on the number, total biomass or body size of any of the other common prey species in the stomachs of plaice or dab (electronic supplementary material, table A3).

## 4. Discussion

The results presented in this study clearly show that bottom trawling reduced the overall abundance and biomass of available prey for two commercial flatfish, plaice and dab. However, concurrent declines in fish abundance at more highly trawled sites [1] meant that the ratio of prey biomass to fish biomass was not reduced. The results also demonstrate that even at heavily trawled sites, prey consumption and the total energy content of stomach contents of these fish were maintained. The results therefore show, for the first time, that fish living in highly trawled areas are still able to maintain food intake when the composition and quantity of their food supply is changed as a result of chronic bottom trawling. Although these results are specific to plaice and dab, they provide an important advance on previous work, which has often suggested that a reduction in overall prey availability as a consequence of trawling leads to reduced food intake by resident demersal fish populations [1,7,11] and a subsequent reduction in fish body condition [1,7]. By contrast, our results show that the observed reduction in body condition of plaice with trawling [1] was not the effect of a lowered food intake. Instead, changes in the diet of plaice were observed that are likely to be linked to potential reductions in foraging efficiency of this species. Such dietary changes were not noted for dab along the trawling gradient analysed and this species demonstrated no reduction in condition at high trawl frequencies in our study area.

The analysis of the stomach contents of plaice revealed that two major prey taxa, *Nephtys* spp. (Polychaeta) and *A. alba* (Bivalvia), were preferentially selected and responded significantly to trawling frequency. The high-energy prey *Nephtys* spp. decreased in size with increased trawling in the stomachs of plaice and in the environment. No changes in the abundance of *Nephtys* spp. were noted in the stomachs or environment. The matching trends in the size of *Nephtys* spp. in the stomachs and in the environment suggest that plaice were not actively searching or foraging for smaller *Nephtys* spp. at higher trawled sites. The abundance of the less energy-rich bivalve *A. alba* showed no significant change in abundance in the stomachs of plaice or in the environment. The total biomass of this species did, however, increase in the stomachs at highly trawled sites. A separate analysis showed that this suggested increase in size of ingested *A. alba* with trawling was detected in the stomachs but was not reflected in the environment, and therefore could represent an active selection by plaice for larger *A. alba* individuals at higher trawl frequencies. Feeding on such specific prey items potentially reduces energetic gains when compared with similar biomasses of higher energy-rich prey such as *Nephtys* spp. The lower condition of plaice at highly trawled sites [1] may therefore be related to increased energetic costs of targeting the deep burrowing [37], low-energy-content *A. alba* [33], with more time and energy spent swimming, searching and foraging for buried food items [38]. This supports suggestions by Smith *et al.* [10] that with increased prey availability comes a probable reduction in efforts of prey detection by benthivorous fish and overall reductions in energy expenditure.

Although trawling reduced the overall biomass of prey available to plaice and dab, a concomitant decline in the biomass of the fish [1], probably related to fish mortality and removal by the *Nephrops* fishery as bycatch, meant that the biomass of prey available per individual fish did not decline. This suggests that fish at highly trawled sites theoretically had levels of prey per fish similar to less trawled areas. However, as the overall prey density at these sites is lower individual fish are likely to require increased searching effort during foraging bouts. Therefore, rather than reduced feeding, increased foraging effort is a potentially important mechanism that could affect the body condition of fish remaining in areas of low prey density, following chronic trawl events. In order to robustly test such a hypothesis, additional work would be needed involving the *in situ* tracking of fish in areas of different trawling activity, as well as laboratory-based experiments to measure the extent to which differences in prey density cause changes in foraging effort and behaviour, and affect overall energy gains from prey capture.

Dab are a widely distributed flatfish species with high levels of exploitation and bycatch mortality [39], but generally large and stable population sizes [40,41]. Their resistance to exploitation may in part be the result of their feeding strategy. In contrast to plaice, dab feed on a wide-ranging diet of larger and more energy-rich prey items. Feeding on larger individuals may involve increased prey handling time, and therefore increased energy costs [42]. It is, however, likely to provide more feeding opportunities for dab compared with plaice, especially in areas of low prey abundance and density caused by trawling. Dab stomachs contained a high number of crustacean chelipeds, which were totally absent in plaice stomachs. The crustacean species fed upon by dab are primarily burrowing species, and feeding solely on their appendages may well remove the necessity to spend a lot of energy digging individual prey items out from their burrows. This feeding strategy is likely to lead to a higher energy profit per prey capture compared with that of plaice, especially considering crustaceans are among the most energy-rich prey in the diets of both plaice and dab [33]. As a consequence, dab potentially spend less time foraging than plaice, with more resting periods between foraging bouts, and hence have an overall more energetically favourable foraging strategy [38]. The ability to feed on larger prey items, to higher levels of fullness (also seen in juveniles of the species [26]), and on a range of high-energy content prey items such as crustaceans and their appendages, is likely to maintain high condition in dab at highly trawled sites. Overall, it appears that dab are largely unaffected by trawling as they can readily adapt their diet to trawling-induced disturbance without subsequent reductions in feeding efficiency.

The potential modification of prey resources by trawling-induced disturbance for any benthivorous fish species obviously increases with trawling intensity. The majority of previous investigations into the effects of bottom trawling on fish condition have focused on fish that are targeted by the local fishery [7,11]. Hence, there exists a self-correction feedback loop whereby as fishing intensity increases, target fish numbers decline and so effort declines. The fish in this study, although commercially important, were collected from a trawl ground where they are only a bycatch species. Here, a reduction in the abundance of plaice and dab as fishing intensity increases (as noted by Hiddink *et al.* [1]) will not directly affect fishing effort. This means that the modification of the prey resource of plaice and dab is potentially worse than may be expected for fisheries that target these species and respond to their local abundance. This could have long-term population-level consequences for the local fish populations in the area.

There are a number of important assumptions made in our work, namely that plaice and dab were feeding over areas at which they were caught, that their stomach contents reflect patterns in the local prey environment and related directly to individual condition, and that grab sampling gave an accurate representation of the prey community. Foraging theory predicts that predators will move away from areas of poor prey quality [43]. It is, however, unlikely that flatfishes are able to detect gradients in habitat quality across spatial scales necessary to redistribute across our large study area [44]. We therefore suggest that the reduced numbers of both plaice and dab at highly trawled sites is due to local fishery bycatch rather than self-motivated redistribution in search of better habitat. Although relatively little is known about the movement of these flatfish species, tracking studies by Hunter *et al.* [45] showed that plaice hardly moved in June, the month of our study. The evacuation rates of plaice and dab are known to be between 14 h at 15°C and 9.28 h at 16.4°C, respectively [46,47]. Considering a water temperature of 12 ± 1°C (during our survey), it is reasonable to assume that stomach contents did reflect local feeding as fish would need to move from approximately 1 to 2.5 km within 12 h after feeding in order to be caught within an area of different trawl frequency.

The conditions of plaice and dab noted by Hiddink *et al.* [1] were causally related to the feeding of each individual. It should, however, be noted that in highly trawled areas, changes in prey assemblages could combine with other indirect effects of fishing gear to impact on fish condition. These may include encounters with fishing gears causing injury [48], reductions in immune responses [49], increased energy expenditure avoiding trawl gears and associated noise [50,51], increased risks of predation for smaller individuals [50], and reduced visual and chemosensory acuity leading to increased difficulties in prey encounter and capture [52]. As the abundance of both plaice and dab decreased with increased trawling [1], it was assumed that the behavioural responses to living in a stressful trawl-disturbed environment acted equally on plaice and dab. Further in-depth study would, however, be required to test these assumptions and elucidate if any of these additional factors have a significant bearing on the foraging capability and resultant condition of either plaice or dab.

The extent to which grab sampling gives an accurate representation of the prey community available to plaice and dab has an important impact on the confidence in our conclusions and all similar work. It is clear that the numbers of deeply buried or fast-moving crustacean species, such as some amphipod species or burrowing crustaceans (e.g. *G. rhomboides*, *J. nocturna* and *C. subterranea*), which may be able to avoid the jaws of the grab, could have been underestimated. If these types of prey are more abundant than we estimate, and because the abundance of these species was not affected by trawling (possibly also because they are deep living), the abundance of dab prey may not actually have declined with trawling. This could be an alternative explanation for the lack of a response of dab stomach contents to trawling.

We demonstrate that even in areas showing significant reductions in overall local prey availability following chronic trawling activity, resident fish populations are able to maintain consistent levels of feeding. This contradicts the common suggestion that reduced prey availability leads to declines in feeding (and consequently body condition) of resident populations. Dietary changes in plaice observed along the trawling gradient, together with a potential decrease in foraging efficiency, linked to changes in size and prey quality, were identified as the most plausible causes for the negative trends in plaice condition observed in our study area by Hiddink *et al.* [1]. The generally low-energy diet of plaice and overall lower levels of stomach fullness, combined with an apparent inability to target other more energy-rich prey, make fish with specialized feeding strategies (like plaice) more vulnerable than generalist feeders (like dab) to changes in prey communities caused by trawling. Understanding which fish species or life stages are prone to the negative effects of bottom trawling as well as the mechanisms by which fish foraging and resultant condition may be reduced will be important in the formulation of mitigation measures for fisheries management.

## Supplementary Material

Figure A1

## Supplementary Material

Figure A2

## Supplementary Material

Table A1

## Supplementary Material

Table A2

## Supplementary Material

Table A3

## Data accessibility

All supporting data are available from CEFAS via DAPSTOM (integrated database and portal for fish stomach records). This is an ongoing initiative to digitize and make available fish stomach content records. Data can be searched for by predator (plaice or dab) or by prey species (any of the infaunal prey found in the stomachs in this study). Data can be searched for by species, year or ICES subdivision area (in this case 2009, Irish Sea (VII A)). See http://www.cefas.defra.gov.uk/our-science/fisheries-information/fish-stomach-records.aspx. The DAPSTOM database is also available through http://www.data.gov.uk/dataset/dapstom.
