# Supplementary figures and images for: Effects of bottom trawling on fish foraging and feeding
Source: Proc Biol Sci. 2015 Jan 22;282(1799):20142336. doi: 10.1098/rspb.2014.2336 (PMC4286059; doi:10.1098/rspb.2014.2336)

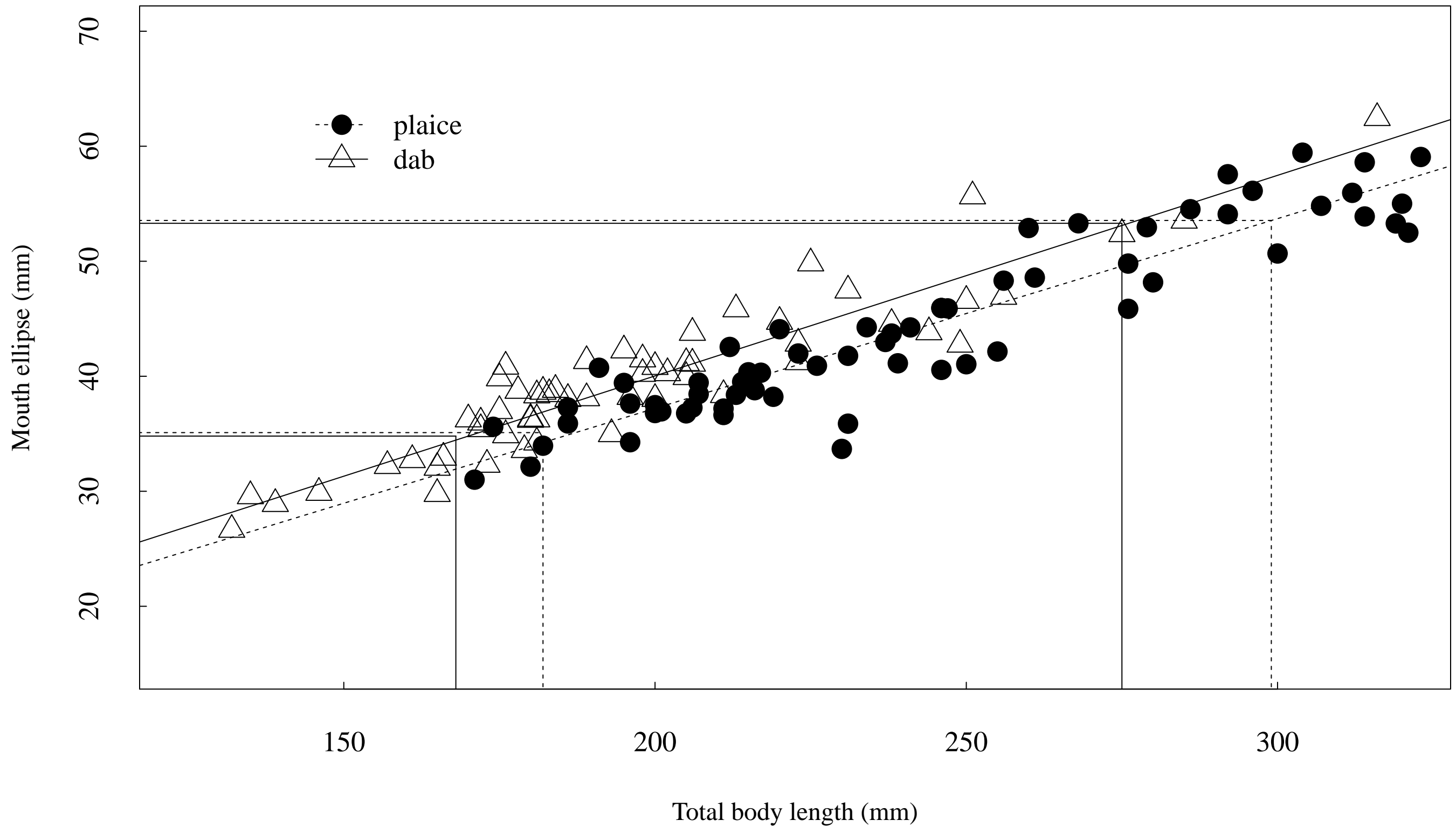

Supplement: Figure A1 [file rspb20142336supp1.pdf]

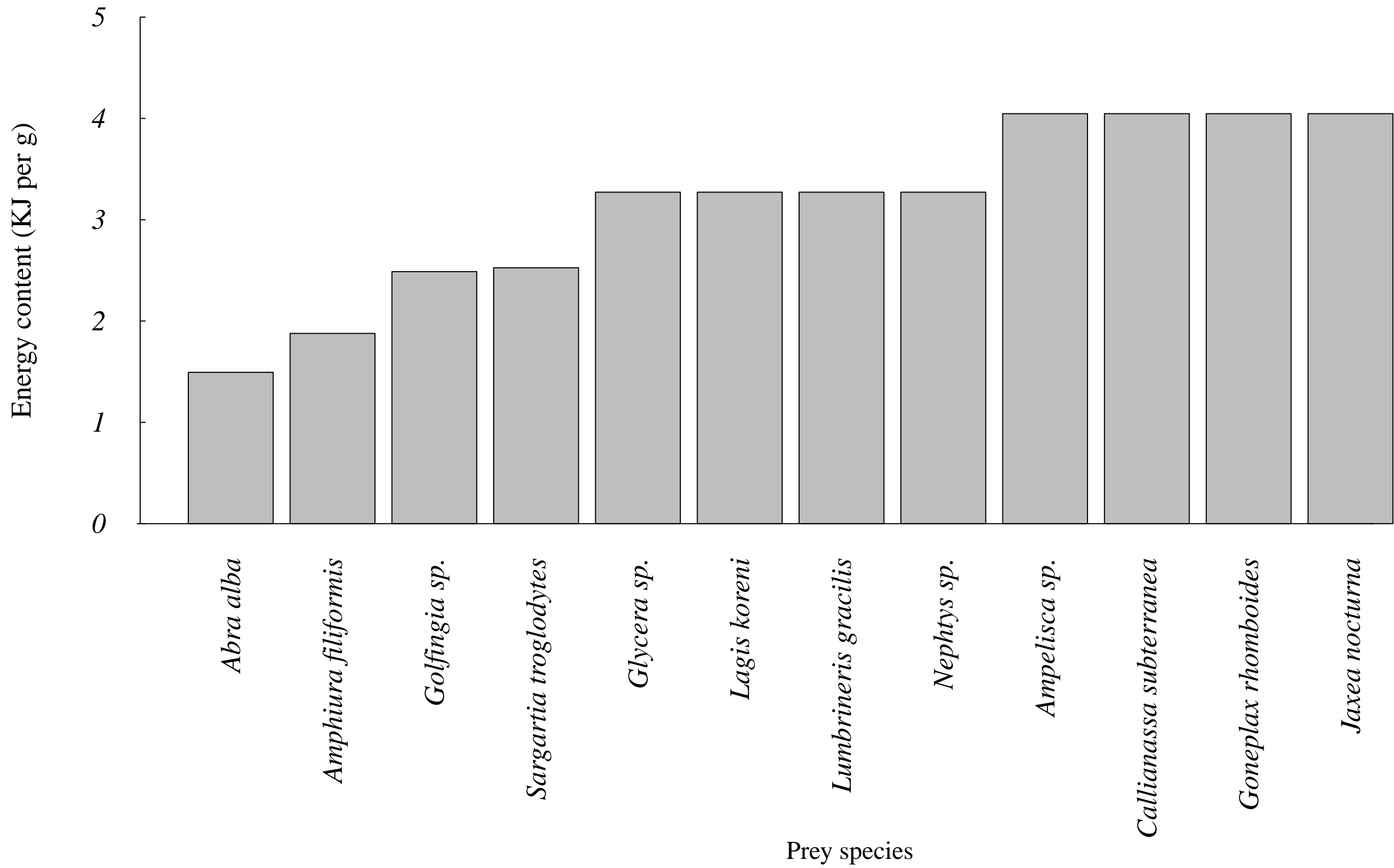

Supplement: Figure A2 [file rspb20142336supp2.pdf]
