# Supplementary material for: Effects of bottom trawling on fish foraging and feeding
Source: Proc Biol Sci. 2015 Jan 22;282(1799):20142336. doi: 10.1098/rspb.2014.2336 (PMC4286059; doi:10.1098/rspb.2014.2336)
Supplement: Table A1 [file rspb20142336supp3.docx]

| Trawl frequency yr^-1^ | Station | Dab nº | Plaice nº |
| --- | --- | --- | --- |
| 0.5 | H | 32 | 55 |
| 2.7 | T | 50 | 47 |
| 3.6 | R | 28 | 19 |
| 5.8 | C | 33 | 13 |
| 6 | S | 35 | 17 |
| 7 | P | 41 | 28 |
| 7.3 | B | 40 | 38 |
| 7.7 | L | 28 | 28 |
| 7.8 | I | 36 | 1 |
| 8.5 | F | 15 | 11 |
| 8.8 | D | 47 | 27 |
| 9.4 | G | 47 | 15 |
| 10.2 | M | 52 | 25 |
| 10.5 | E | 46 | 40 |
| 11.9 | O | 45 | 50 |

Table A1. Total numbers of plaice and dab stomachs sampled per trawl frequency with corresponding site letters.
