# Supplementary material for: Effects of bottom trawling on fish foraging and feeding
Source: Proc Biol Sci. 2015 Jan 22;282(1799):20142336. doi: 10.1098/rspb.2014.2336 (PMC4286059; doi:10.1098/rspb.2014.2336)
Supplement: Table A2 [file rspb20142336supp4.docx]

Table A2. Results of OLS regression analyses (Log_10_ total biomass in the environment at each trawl site against trawl frequency (df =14)) for the top 90% (by abundance) of infaunal species ordered by slope. Θ denotes presence in the diet, § denotes the common species (≥10) in the diet and numbers in parentheses denote order of selection (calculated as Chesson’s index see figure 2; those in bold were selected for preferentially). * indicates significance after FDR correction.

| **Species** | **Plaice** | **Dab** | **Slope** | ***R²*** | ***F*** | ***P*** |
| --- | --- | --- | --- | --- | --- | --- |
| *Amphiura filiformis* | §(8) | §(8) | -0.073 | 0.528 | 14.53 | 0.002* |
| *Spisula subtruncata* |  |  | -0.017 | 0.377 | 7.864 | 0.045 |
| ***Glycera sp.*** | **§(2)** | **§(6)** | **-0.02** | **0.39** | **8.464** | **0.012*** |
| *Scalibregma inflatum* |  | Θ | -0.007 | 0.061 | 0.844 | 0.375 |
| *Mysia undata* |  |  | -0.006 | 0.205 | 3.359 | 0.09 |
| *Lagis koreni* | §(5) | Θ | -0.004 | 0.539 | 15.21 | 0.002* |
| ***Nephtys sp.*** | **§(3)** | **§(7)** | **-0.003** | **0.041** | **0.562** | **0.467** |
| *Phoronis sp.* |  | Θ | -0.003 | 0.527 | 14.51 | 0.002* |
| *Edwardsia clapparedii* |  | Θ | -0.002 | 0.572 | 17.39 | 0.001* |
| *Melinna elisabethae* | Θ | Θ | -0.002 | 0.391 | 8.298 | 0.013* |
| *Magelona alleni* | Θ |  | -0.001 | 0.188 | 3.001 | 0.107 |
| *Mysella bidentata* |  |  | -0.001 | 0.397 | 8.554 | 0.012 |
| ***Abra alba*** | **§(1)** | **§(4)** | **0** | **0.004** | **0.046** | **0.833** |
| *Ampelisca sp.* | Θ | §(5) | 0 | 0.049 | 0.664 | 0.43 |
| ***Callianassa subterranea*** | **Θ** | **§(2)** | **0** | **0.032** | **0.432** | **0.522** |
| *Corbula gibba* |  |  | 0.001 | 0.374 | 7.754 | 0.015* |
| *Cylichna cylindracea* |  |  | 0 | 0.206 | 3.364 | 0.09 |
| *Golfingia sp.* | §(6) | Θ | 0 | 0.001 | 0.008 | 0.932 |
| *Lumbrineris gracilis* | §(4) | Θ | 0 | 0.083 | 1.178 | 0.298 |
| *Polydora sp.* |  |  | 0 | 0.043 | 0.589 | 0.456 |
| *Prionospio sp* |  |  | 0 | 0.016 | 0.206 | 0.657 |
| *Notomastus sp.* |  |  | 0.003 | 0.043 | 0.585 | 0.458 |
| ***Jaxea nocturna*** | **§(7)** | **§(3)** | **0.005** | **0.188** | **3.008** | **0.106** |
| ***Goneplax rhomboides*^α^** |  | **§ (1)** | **-** | **-** | **-** | **-** |

^α^Occurrences of *G. rhomboides* at less than 4 sites mean OLS regression was not possible for this species.
