# Supplementary material for: Effects of bottom trawling on fish foraging and feeding
Source: Proc Biol Sci. 2015 Jan 22;282(1799):20142336. doi: 10.1098/rspb.2014.2336 (PMC4286059; doi:10.1098/rspb.2014.2336)
Supplement: Table A3 [file rspb20142336supp5.docx]

Table A3. Results of OLS regression analyses of the body size, total biomass and abundance in stomachs against trawl frequency for the common prey species of plaice (a) and dab (b). Results in bold are plotted in figure 6a, b, c. * indicates significance after FDR correction.

(a)

| **Species** | **Body size** | | | | **Total biomass** | | | | **Abundance** | | | |
| --- | --- | --- | --- | --- | --- | --- | --- | --- | --- | --- | --- | --- |
|  | **Slope** | ***R*²** | ***F*_1,13_** | ***P*** | **Slope** | ***R*²** | ***F*_1,13_** | ***P*** | **Slope** | ***R*²** | ***F*_1,13_** | ***P*** |
| ***Abra alba*** | **0.002** | **0.376** | **5.988** | **0.034** | **0.039** | **0.63** | **20.82** | **<0.001*** | **0.020** | **0.095** | **1.256** | **0.284** |
| *Glycera sp.* | <0.001 | <0.001 | 0.069 | 0.989 | -0.016 | <0.001 | <0.001 | 0.969 | -0.036 | 0.001 | 0.011 | 0.919 |
| ***Nephtys sp.*** | **-0.004** | **0.477** | **12.83** | **0.004*** | **-0.045** | **0.10** | **1.343** | **0.269** | **-0.004** | **0.384** | **7.477** | **0.018** |
| *Lumbrineris gracilis* | 0.002 | 0.128 | 0.209 | 0.989 | 0.005 | 0.063 | 0.806 | 0.387 | 0.07 | 0.014 | 0.174 | 0.684 |
| *Lagis koreni* | -0.004 | 0.020 | 0.285 | 0.603 | -0.017 | 0.089 | 1.175 | 0.3 | -0.053 | 0.016 | 0.200 | 0.662 |
| *Golfingia sp.^§^* | - | - | - | - | - | - | - | - | -0.028 | 0.013 | 0.160 | 0.696 |
| *Jaxea nocturna* | -0.004 | 0.017 | 0.208 | 0.656 | -0.003 | <0.001 | 0.006 | 0.936 | -0.001 | <0.001 | 0.001 | 0.978 |
| *Amphiura filiformis* | -0.002 | 0.16 | 2.279 | 0.157 | -0.02 | 0.131 | 1.807 | 0.204 | -0.108 | 0.01 | 0.123 | 0.732 |

*^§^accurate estimates* *body size for Golfingia sp. were not possible due to digestive state.*

(b)

| **Species** | **Body size** | | | | **Total biomass** | | | | **Abundance** | | | |
| --- | --- | --- | --- | --- | --- | --- | --- | --- | --- | --- | --- | --- |
|  | **Slope** | ***R*²** | ***F*_1,14_** | ***P*** | **Slope** | ***R*²** | ***F*_1,14_** | ***P*** | **Slope** | ***R*²** | ***F*_1,14_** | ***P*** |
| *Goneplax rhomboides* | -0.078 | 0.137 | 2.068 | 0.174 | -0.129 | 0.121 | 1.787 | 0.204 | 0.006 | 0.044 | 0.605 | 0.451 |
| *Callianasa subterranea* | 0.006 | 0.004 | 0.047 | 0.832 | 0.092 | 0.023 | 0.30 | 0.593 | <0.001 | 0.023 | 0.307 | 0.589 |
| *Jaxea nocturna* | -0.007 | 0.003 | 0.034 | 0.857 | 0.112 | 0.077 | 1.078 | 0.318 | 0.052 | 0.031 | 0.412 | 0.532 |
| *Abra alba* | -0.001 | 0.003 | 0.041 | 0.843 | 0.002 | 0.005 | 0.063 | 0.806 | 0.017 | 0.025 | 0.331 | 0.575 |
| *Ampelisca sp.* | <0.001 | 0.041 | 0.554 | 0.47 | 0.001 | 0.013 | 0.177 | 0.68 | 0.018 | 0.027 | 0.366 | 0.556 |
| *Glycera sp.* | -0.004 | 0.003 | 0.036 | 0.851 | -0.027 | 0.014 | 0.188 | 0.672 | 0.038 | 0.014 | 0.188 | 0.671 |
| *Nephtys sp.* | 0.001 | 0.0211 | 0.139 | 0.715 | 0.001 | <0.001 | <0.001 | 0.997 | 0.075 | 0.019 | 0.255 | 0.622 |
| *Amphiura filiformis* | -0.008 | 0.137 | 2.061 | 0.175 | -0.042 | 0.358 | 6.697 | 0.024 | -0.015 | 0.091 | 1.197 | 0.295 |
| *Sargartia troglodytes^§^* | - | - | - | - | - | - | - | - | 0.005 | 0.015 | 0.192 | 0.669 |

*^§^accurate estimates* *body size for S. troglodytes were not possible due to digestive state.*
